# Mystery and Fetichism

**Published:** 1914-04

**Authors:** 


### Mystery and Fetichism

One of the most disgusting—and one might almost say, amusing—phases of sexual perversion is fetichism. Yet, it is common in quite an innocent form. Consider the literature of a certain type, that has crystalized about a lady's glove, her handkerchief and, with only mild impropriety, about even her garter, stocking or corset. Prevailing fashions and customs have tended to remove the mystery from femininity in many ways. Romantic fetichism is apparently not so prevalent as formerly. Who, for instance, could become sentimental about an apparatus of elastic and metal, whose mechanic advantages are illustrated in street cars; or about undergarments which are pictured every day in the papers, with prices, and many of which are only partially concealed beneath the outer covering which represents what used to be called a dress? A good deal of the finer chivalry has been lost, but, with it, there has passed a lot of sentiment and immature eroticism. Perhaps the somewhat startling candor of the present will establish new standards of propriety and, after the readjustment, we will find that our social life has become more sensible and wholesome.

